# Can individuals with a significant risk for cardiovascular disease be adequately identified by combination of several risk factors? Modelling study based on the Norwegian HUNT 2 population

**DOI:** 10.1111/j.1365-2753.2008.00962.x

**Published:** 2009-02

**Authors:** Halfdan Petursson, Linn Getz, Johann A Sigurdsson, Irene Hetlevik

**Affiliations:** 1 Research Fellow and Medical Student, Department of Family Medicine, University of Iceland, Solvangur Health CentreIS-220 Hafnarfjördur, Iceland; 3 Professor, Department of Family Medicine, University of Iceland, Solvangur Health CentreIS-220 Hafnarfjördur, Iceland; 2 Associate Professor, Research Unit of General Practice, Department of Public Health and General Practice, Norwegian University of Science and Technology (NTNU)Trondheim, Norway; 4 Professor, Research Unit of General Practice, Department of Public Health and General Practice, Norwegian University of Science and Technology (NTNU)Trondheim, Norway

**Keywords:** cardiovascular risk estimation, clinical practice, guidelines, modelling study, prehypertension, preventive medicine

## Abstract

**Rationale, aims and objectives:**

Clinicians are generally advised to consider several risk factors when evaluating patients' cardiovascular disease (CVD) risk. Our aim was to study whether combined assessment of five traditional risk factors might help doctors demarcate a relatively distinct and manageable group of high-risk individuals. We selected five modifiable risk factors and estimated the proportion of a well-defined population with ‘unfavourable’ levels of at least two of them, as defined by four internationally renowned guidelines. The impact of including so-called ‘prehypertension’ among the risk factors was specifically addressed, and the results are discussed in a wider perspective.

**Material and methods:**

Guideline implementation was modelled on data from a cross-sectional Norwegian population study comprising 62 104 adults aged 20–79 years (The Nord-Tröndelag Health Study 1995–7). Total, age- and gender-specific point prevalences of individuals with zero, one, two, three or more factors, in addition to established disease, were calculated.

**Results:**

One single CVD risk factor was exhibited by 12.4% of the population; two factors by 21.5%; and three or more by 49.7%. Established CVD or diabetes mellitus was reported by 12.5%. In total, 83.7% of the population exhibited a risk or disease profile with at least two factors, if prehypertension was included.

**Conclusions:**

If guideline recommendations are literally applied, as many as 84% of adults in Norway could exhibit two or more CVD or risk factors and thus be considered in need of individual, clinical attention. This challenges the widely held presumption that ‘the net will close’ around a manageable group of individuals-at-risk if several risk factors are jointly considered. As the finding of this study arises in one of the world's most long- and healthy-living populations, it raises several practical as well as ethical questions.

## Introduction

Cardiovascular disease (CVD) is currently the major cause of death in the Western world, including Europe (see e.g. http://www.heartcharter.eu). Mortality from CVD has, however, declined substantially since the 1970s [1], and many Western populations appear to be undergoing the resolution phase of a so-called 20th-century epidemic of CVD. This epidemic reportedly began in the 1930s and peaked in the 1950s and 1960s. The epidemic's impact on medical thought and practice has been profound, but still, the reasons behind the rise and fall of CVD in the 20th-century are far from clear [2]. In view of the new ‘epidemics’ of obesity and diabetes, there is a widespread concern that the burden of CVD will start to rise again.

Large-scale medical searches for factors that could predict future heart disease began shortly after World War II with the establishment of the US Framingham study. Around 1960, hypertension, hypercholesterolemia and smoking were singled out as the three most apparent risk factors for ischaemic heart disease. Since then, increasingly detailed knowledge regarding the impact of numerous measurable and potentially modifiable biological risk factors and risk markers has been published [2]. Development and marketing of tolerable and presumably safe antihypertensive and lipid lowering drugs have contributed much to the immense interest in CVD prevention among researchers and clinicians.

In 1962, the World Health Organization published the first international report on the importance of blood pressure control [3]. After this milestone publication, subsequent generations of clinical recommendations and guidelines regarding blood pressure as well as a steadily increasing number of other measurable risk factors have been released on both sides of the Atlantic [4–13]. With time, the thresholds for clinical intervention on the basis of single risk factors have been lowered several times. Redefinition of a risk factor cut-off point inevitably leads to a corresponding change in the number of individuals that will be categorized as ‘at risk’ and in need of clinical attention [14,15]. In 2004, our research group documented that implementation of the 2003 European guidelines on CVD prevention could label as much as 76% of a general Norwegian population aged 20 years and older, and 90% of individuals aged 50 years and older, as having unfavourably high cholesterol and/or blood pressure levels [15]. Arising in the context of one of the world's longest-living and healthiest-living populations [16], this finding was highlighted in an editorial in the *British Medical Journal*[17]. A heated debate followed on the *BMJ* website [17]. Key authors of the European guidelines entered the debate and stated that the focus on single risk factors represented a startling lack of understanding, and argued that the European guidelines would not lead to medicalization of whole populations since CVD risk should in practice be evaluated on the basis of a *combined risk factor estimate*. This advice was (and still is) in line with mainstream clinical recommendations for good practice [7,9,13]. The crucial question, however, is how well-combined risk evaluation strategies actually work in practice. Will they, as intended, aid clinicians to define a relatively well-demarcated and manageable high-risk group who can be targeted for further follow-up?

### Formal, combined risk calculators

From the viewpoint of the practicing clinician, there are in principle two ways of performing a combined CVD risk evaluation. The first method involves a formal ‘risk prediction algorithm’ or ‘calculator’ developed on the basis of epidemiological outcome data. The level of a given individual's conventional risk factors (modifiable, such as blood pressure, cholesterol and smoking; and unmodifiable, i.e. age and gender) will be fed into the algorithm and result in a computed estimate of risk (for disease events or mortality). The first algorithm of this kind was based on the US Framingham study [18]. The Framingham model has later been updated and adjusted for use both in the United States and in Europe. In 2003, a European risk system called SCORE (Systematic Coronary Risk Evaluation) was launched, based on data from 12 European cohort studies recruited from the 1970s on [9,19,20]. In 2003 and 2007, European guidelines on CVD prevention based on the SCORE system have been published [9,12].

Although a combined risk approach may often represent an adequate, general approach to CVD risk evaluation, the clinical implementation of combined CVD risk algorithms has encountered problems. It has been well documented in a variety of settings that both the US Framingham and the European SCORE models may lead to significant overestimation of risk [9,12,21–23]. In 2005, our research group went on to document that implementation of the SCORE risk system, as outlined in the 2003 European guidelines on CVD prevention, could also label a majority of the above-mentioned Norwegian population as in need of ‘maximal clinical attention’ due to high *combined* risk [21].

The main reason for the risk calculators' tendency to statistically overestimate risk is most likely the previously mentioned epidemiological decline in CVD incidence leading to a so-called ‘retrospective risk bias’[12,21]. To tackle this dilemma, guideline authors recommend that risk calculators be calibrated against national data. But even in the presence of mathematically valid risk calculators, the size of the high-risk group might still be so large as to represent a major challenge.

### Consideration of multiple risk factors

Clinicians who do not have access to a calibrated and validated risk calculator for use in their local setting are likely to apply a more straightforward approach to combined risk evaluation. The typical case would involve an otherwise healthy person who presents with a moderately increased blood pressure, or a moderately increased cholesterol. In this situation, the doctor may or should, according to guidelines, consider the level of other risk factors before deciding on further action. The presence of more than one elevated risk factor (beyond the currently recommended cut-off point) would strengthen the argument for clinical intervention, while absence of additional risk factors might justify a more expectant approach.

The aim of the present study was to examine the practical usefulness of the latter CVD risk evaluation method from a clinical viewpoint. We selected five modifiable risk factors, and with reference to recommended cut-off points in four internationally renowned guidelines, studied what proportions of a given Norwegian population would exhibit ‘unfavourable’ levels of at least two of them. In our analysis, we specifically addressed the emerging risk (or literally pre-risk) factor called ‘prehypertension’ (120–139/80–89 mmHg) which has been included in some guidelines since 2003 [8]. Other more recent guidelines avoid using this ambiguous, medical term. They label a blood pressure level of 120–129/80–84 mmHg as ‘normal’, but indicate that it is not optimal. The level 130–139/85–89 mmHg is termed ‘high normal’[9,11,13]. Whatever terminology used, blood pressure in the ranges 120–139/80–89 have attracted increasing attention as a risk factor for CVD among researchers and clinicians in the field of CVD prevention [24–26].

## Materials and methods

Based on data from a large and well-organized Norwegian population study: the Nord-Tröndelag Health Study 1995–7 (HUNT 2 study) [27], we estimated the proportions of the population who would exhibit unfavourable combinations of risk factors, if evaluated according to the following CVD prevention recommendations: The guidelines from the American Heart Association (AHA) [7], the European Society of Cardiology [9,12], the UK National Institute for Clinical Excellence [10] and the US Joint National Committee (seventh report, JNC 7) [8], respectively. The guidelines' recommended cut-off points are listed in Table 1.

**Table 1:** Overview of the selected risk factors' cut-off points in four clinical guidelines on CVD: the American Heart Association (AHA), European guidelines on CVD prevention in clinical practice (EUR), National Institute of Clinical Excellence (NICE) and the Seventh Report of the Joint National Committee on Prevention, Detection, Evaluation and Treatment of High Blood Pressure (JNC 7)

Guideline	Regular assessment by health care providers	Medical treatment or follow-up by health care providers
AHA	BP ≥ 130/80 mmHg	BP ≥ 140/90 mmHg
	BMI ≥ 25.0 kg m^−2^	
	Waist circumference	
	Men >102 cm	
	Women >88 cm	
	Smoking	
	Family history of CVD	
EUR	Cholesterol ≥ 5 mmol L^−1^	BP ≥ 140/90 mmHg
	BMI ≥ 25.0 kg m^−2^	Cholesterol > 8 mmol L^−1^
	Waist circumference	
	Men >102 cm	
	Women >88 cm	
	Smoking	
	Family history of CVD	
NICE	BP > 140/90 mmHg	BP ≥ 160/100 mmHg
	Smoking	
JNC 7	BP ≥ 120/80 mmHg	BP ≥ 140/90 mmHg

BMI, body mass index; BP, blood pressure; CVD, cardiovascular disease.

When modelling the implementation of guidelines, we started by identifying individuals with self-reported myocardial infarction, stroke, angina pectoris or on-going antihypertensive treatment. Patients with diabetes mellitus (both types I and II) were also included here, as current guidelines recommend close CVD surveillance for these patients. We categorized these people as having ‘established disease’ and thus as eligible for clinical attention irrespective of current risk factor levels. Subsequently, all remaining individuals were categorized as ‘below’ or ‘above’ the recommended limits for the following measured and calculated CVD risk variables: blood pressure, total serum cholesterol, waist circumference and body mass index (BMI), and daily smoking. A family history of premature CVD was also considered as a risk factor.

### The HUNT 2 population study

The HUNT 2 study was designed to investigate the significance of biomedical risk factors. Its design and methods have been described in detail elsewhere [27]. The overall participation rate in HUNT 2 was 76% among women and 67% among men (for both sexes combined 20–29 years: 49%; 30–39 years: 68%; 40–49 years: 77%; 50–59 years: 81%; 60–69 years: 86%). The present study is based on data from all participants aged 20–79 years (in total 62 104 individuals, 29 288 males and 32 816 females; see Table 2). The HUNT 2 population has been considered relatively representative for the total Norwegian population regarding demography, socioeconomic factors, morbidity and mortality [27].

**Table 2:** Participants in the HUNT 2 (1995–7) study according to age and gender

Age groups	Men	Women	Total
20–24	1 761	2 156	3 917
25–29	2 163	2 561	4 724
30–34	2 579	2 917	5 496
35–39	2 820	3 207	6 027
40–44	3 161	3 478	6 639
45–49	3 334	3 566	6 900
50–54	3 064	3 314	6 378
55–59	2 333	2 461	4 794
60–64	2 113	2 292	4 405
65–69	2 232	2 418	4 650
70–74	2 134	2 382	4 516
75–79	1 594	2 064	3 658
Total	29 288	32 816	62 104

In the HUNT 2 survey, blood pressure was measured on persons in seated position by specially trained personnel using a Dinamap 845XT based on oscillometry. Cuff size was adjusted after measuring the arm circumference, and blood pressure was recorded as the mean values of the second and third of three measurements performed consecutively at the same visit. Total cholesterol was measured by an enzymatic colorimetric cholesterolesterase method [27]. Height was measured to the nearest 1.0 cm and weight to the nearest 0.5 kg and BMI was calculated as kg m^−2^.

In the present analysis, smoking was defined as daily smoking of cigarettes, cigars or pipe. Family history of CVD was defined as first-degree relatives (parents or siblings) with myocardial infarction before the age of 60 or stroke at any age.

For international comparison, prevalence rates were also calculated according to the European and World age standardization (Table 3) [28].

**Table 3:**
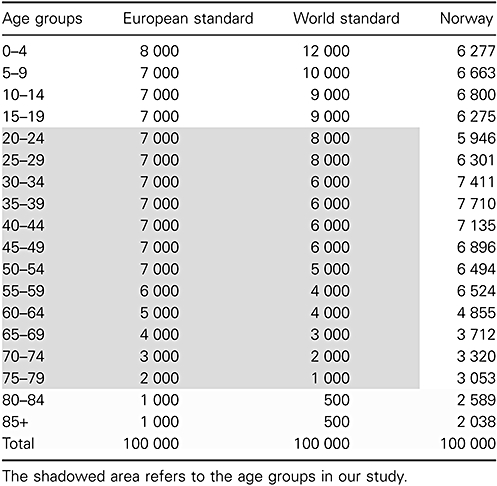
Standardized age distribution of inhabitants in Europe and the world, as well as age distribution of the Norwegian population in 2005

The spss statistical package, version 15.0 (SPSS Inc., Chicago, IL, USA), was used for statistical frequency analyses. All surveys in HUNT 2 were approved by the Norwegian Data Inspectorate and the Regional Committee for Ethics in Medical Research.

## Results

The prevalence numbers for each separate risk factor is shown in Table 4. Unfavourably, high blood pressure (including both hypertension and prehypertension) is the single most prevalent risk factor for which medical attention would be recommended, followed by total cholesterol. In many instances, the prevalence of the risk factors varied significantly when calculated according to the European and World age standardization.

**Table 4:** Prevalence and 95% CIs of CVD and selected CVD risk factors in the HUNT 2 (1995–7) study as well as calculated prevalence according to the European and World age standardizations

		Age standardized prevalence percentage				
Risk factors and diseases	Prevalence percentage (absolute numers)	Europe	95% CI	World	95% CI	Number missing
Hypertension*	43.2 (26 687/61 844)	38.6	38.2–38.9	35.2	34.8–35.6	260
Prehypertension†	37.8 (23 390/61 871)	40.3	40.0–40.7	42.1	41.7–42.5	233
Cholesterol ≥ 5 mmol L^−1^	75.8 (46 935/61 929)	72.1	71.7–72.5	68.7	68.4–69.1	175
Body fat						
BMI = 25.0–29.9	43.3 (26 718/61 667)	42.2	41.8–42.6	41.1	40.7–41.5	437
BMI ≥ 30.0	16.6 (10 217/61 667)	15.6	15.3–15.9	14.8	14.5–15.1	437
Waist obesity‡	18.4 (11 291/61 320)	16.6	16.4–16.9	15.4	15.1–15.7	784
Smoking	33.7 (18 288/54 244)	33.6	33.2–34.0	33.5	33.1–33.9	7860
Close relatives§ with CVD	32.7 (17 797/54 437)	30.3	29.9–30.6	28.1	27.7–28.5	7667
Established disease and/or on preventive treatment	15.8 (9 754/61 769)	12.5	12.3–12.8	10.5	10.3–10.8	335
Myocardial infarction	2.9 (1 781/61 847)	2.2	2.1–2.3	1.8	1.7–1.9	257
Stroke	1.6 (1 006/61 804)	1.2	1.1–1.3	1.0	0.9–1.1	300
Angina pectoris	4.4 (2 702/61 801)	3.2	3.1–3.3	2.6	2.5–2.7	303
Diabetes	2.7 (1 653/61 863)	2.1	2.0–2.3	1.8	1.7–2.0	241
Antihypertensive treatment	10.4 (6 421/61 845)	8.3	8.1–8.5	7.0	6.8–7.2	259
One or more of the above	97.6 (60 051/61 522)	97.0	96.9–97.1	96.5	96.3–96.6	582
One or more of the above except prehypertension	94.8 (57 727/60 872)	93.3	93.1–93.5	92.0	91.8–92.2	1232

* Hypertensinon defined as ≥140/90 mmHg or on antihypertensive treatment.

† Prehypertension defined as blood pressure 120/80–139/89 mmHg without antihypertensive treatment.

‡ Waist circumference: men >102 cm, women >88 cm.

§ First-degree relatives with a family history of myocardial infarction before the age of 60 or stroke at any time.

BMI, body mass index; CI, confidence interval; CVD, cardiovascular disease.

About 98% of the population had at least one of the risk factors (or established disease conditions) in question. If ‘prehypertension’ was excluded, and only hypertension was considered, this number remained almost unchanged, or 95% (Table 4).

**Figure 1:**
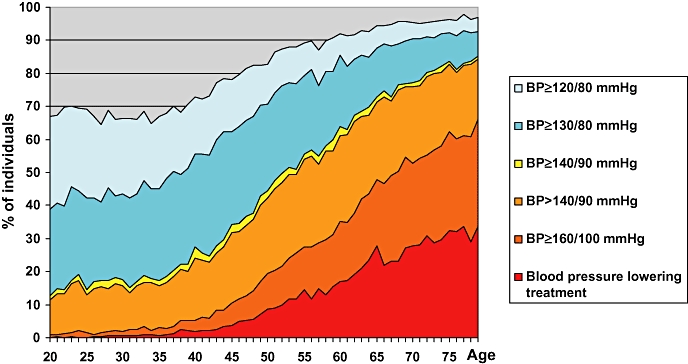
Point prevelence of individuals in each age group (20–79 years) who report being on antihypertensive treatment or whose measured blood pressure (BP) values exceed given limits.

Figure 1 illustrates the potential impact of different blood pressure definitions according to the guidelines studied. Even the minimal difference between applying a hypertension definitions of >140/90 mmHg versus ≥140/90 mmHg would affect 0.8–2.5% of the people within each age group. It is evident that prehypertension is present in a considerable proportion of the population. The currently recommended blood pressure cut-off points ≥130/80 mmHg (by the AHA guidelines) and ≥120/80 mmHg (by the JNC 7 guidelines) would have the greatest consequences among people younger than 50 years.

**Figure 2:**
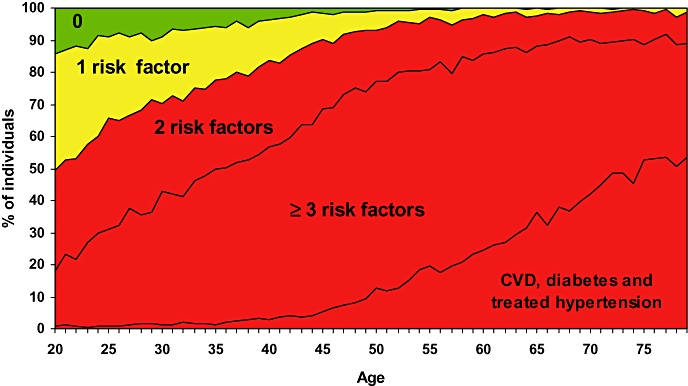
Point prevelence of individuals in each age group (20–79 years) who report established disease (here defined as myocardial infarction, stroke, angina pectoris, diabetes or being on antihypertensive treatment) or one or more of the risk factors studied [i.e. prehypertension or hypertension; high cholesterol; overweight or waist obesity; smoking; or close relatives with cardiovascular diseases (CVD)].

Figure 2 shows the point prevalence of people at different ages who are identified with established CVD, or with three or more, two, one, or zero of the CVD risk factors in question. After age standardization (Europe), it turned out that only 3.9% of the total population would be labelled as free, from both disease and all the above-mentioned risk factors. One single risk factor was exhibited by 12.4% of the population; two risk factors by 21.5%; and three or more factors by 49.7%. Established CVD or diabetes mellitus was reported by 12.5%. In total, 83.7% of the population exhibited a risk or disease profile which involved at least two cardiovascular risk factors.

## Discussion

More than eight of 10 Norwegians may exhibit two or more CVD risk factors – or established disease – and thus be eligible for targeted clinical attention, if current recommendations are interpreted literally. Rather than aiding practicing clinicians to demarcate a reasonable large and manageable high-risk group with respect to further evaluation and follow-up, combination of risk factors appears to inflate the population at risk.

As can be seen from Table 4, prehypertension has a substantial impact on the population at risk, as long as blood pressure is considered in isolation. However, this effect almost vanishes when all five factors are considered jointly. This means that most people with prehypertension also exhibit one or more other risk factors, such as an unfavourably high level of cholesterol.

### Technical strengths and limitations of the present analysis

The HUNT 2 study population is well defined, with high participation rates, and considered fairly representative for Norway as a whole. Compared with other European regions, including regions involved in the MONICA project (third phase, 1992–4) [29], it did not differ significantly with respect to cholesterol levels and smoking habits at the time of data collection. Blood pressure levels were somewhat higher in the HUNT 2 population than in most comparable countries, yet lower than in Finland. We acknowledge that since the data collection in 1995–7, changes may have taken place, both regarding lifestyle and in the distribution of biological disease risk factors in the Norwegian population [30]. We may thereby, like many other investigators in the field of CVD, be introducing a certain retrospective risk bias as we apply population data collected 10 years ago.

With respect to family risk for CVD, the HUNT 2 study enabled us to identify individuals who reported first-degree relative(s) with premature myocardial infarction. Stroke in close relatives, however, was in the HUNT 2 study reported without reference to age. As can be seen from Table 4, this potential source of overestimation has only a minor impact on the resulting risk population.

In this paper, we have deliberately chosen to outline the potential scenario of complete guideline adherence in the area of individual CVD risk assessment. We also chose to include medical surveillance for ‘prehypertension’ in Figs 1 and 2. It can be argued that most guidelines do not go that far in their recommendations as yet. On the other hand, opinion leaders in the medical community appear to be on the brink of accepting the idea, not only of actively monitoring prehypertension, but even to treat it with drugs [24,25]. As an illustration of the increasing focus on the subject, one may note that papers with the words ‘prehypertension’/‘prehypertension’ in the title appeared only sporadically in PubMed until the year 2003 when it suddenly appeared in the title of four papers. In 2004, the number of publications rose to 14; in 2006, 41; and 2007 is likely to see a further increase. It can also be mentioned that Norwegian health authorities in 2003 issued a fee-for-service lifestyle advice scheme ‘Green prescription’ where advice and individual follow-up were indicated even for people with blood pressure in the ‘prehypertensive’ range. It later turned out that the Green prescriptions had low legitimacy among Norwegian general practitioners [31]. One of the reasons may be that the target group was so vast. It has been calculated that literal adherence to the Green prescription initiative could in fact consume half of all consultations in Norwegian primary health care [32].

### A medical ‘vision zero’?

Disease prevention and health promotion are, and should remain, central goals of medicine. It is, however, a major challenge to find a reasonable balance between – on the one hand – biological, specific and risk-oriented approaches to improving health, and – on the other – more general and ‘salutogenetic’ approaches [2]. Definition of relevant and clinically meaningful cut-off levels for individual risk intervention is crucial in this connection. We believe that it can be demoralizing and alienating for primary health care professionals to feel obliged to inform a large majority of the individuals they serve that their current cardiovascular health is not ‘good enough’ according to biomedical standards [32]. The prospect of thus ‘medicalizing’ a large majority of an adult population such as the one in Norway, with one of the world's longest-living and healthy-living populations according to WHO statistics [1,16], evokes several epistemological and ethical challenges [15]. Resource allocation and workload are also among issues that need careful consideration before authoritative clinical recommendations are launched [2,15,21].

We believe the present study serves as a vivid illustration of a piecemeal biomedical empirism that may have become both *too good* and at the same time *not good enough*[33–35]. By ‘too good’, we mean that the statistical impact of traditional, biological risk factors for CVD has now been investigated so extensively that almost every citizen can find empirical arguments for concern related to his or her bodily risk profile. By ‘not good enough’, we mean that a narrow and reductive biological perspective which labels almost every citizen as in need of personalized, medical care, may divert both clinicians' and politicians' attention away from more comprehensive, adequate and sustainable scientific approaches to population health and disease [2].

As primary health care doctors and researchers in two of the richest countries in the Western world, we see it as a duty of the medical community not only to care for the health status of the local individuals we serve, but also to consider our chosen aims and means from a global perspective. We are not convinced that the current trend in direction of an authoritative ‘vision zero’ in the area of CVD prevention represents realistic and sound medicine [2]. Peter Kosso, philosopher of science, has argued that ‘good’ science which really brings humankind forwards tends to have a distinct aesthetical quality to it [35]. If the ‘ethos’ of scientifically based medicine becomes too dominated by surveillance and control of individual's isolated biological factors, down to the lowest levels of risk, it may lose its appeal as an impressive, human endeavour.
